# Efficacy of Stem Cell Therapy in Large Animal Models of Ischemic Cardiomyopathies: A Systematic Review and Meta-Analysis

**DOI:** 10.3390/ani12060749

**Published:** 2022-03-16

**Authors:** Debora La Mantia, Chiara Bernardini, Augusta Zannoni, Roberta Salaroli, Changzhen Wang, Silvia Bencivenni, Monica Forni

**Affiliations:** 1 Department of Veterinary Medical Sciences, University of Bologna, Ozzano dell’Emilia, 40064 Bologna, Italy; debora.lamantia2@unibo.it (D.L.M.); augusta.zannoni@unibo.it (A.Z.); roberta.salaroli@unibo.it (R.S.); changzhen.wang@studio.unibo.it (C.W.); silvia.bencivenni2@unibo.it (S.B.); monica.forni@unibo.it (M.F.); 2 Health Sciences and Technologies—Interdepartmental Center for Industrial Research (CIRI-SDV), Alma Mater Studiorum—University of Bologna, 40126 Bologna, Italy

**Keywords:** stem cells, cell therapy, large animal models, ischemic cardiomyopathies, myocardial infarction

## Abstract

**Simple Summary:**

The present work focuses on stem-cell assessment as a therapeutic approach on cardiovascular diseases, both in terms of safety and efficacy. In particular, this is a systematic review of the relevant literature about the use of stem-cell treatment against acute or chronic ischemic cardiomyopathies in large animal models and a meta-analysis on collected data with regard to the left ventricular ejection fraction (LVEF) as functional parameter. This approach is compliant with the “3Rs” (replacement, reduction and refinement) principle about the use of animal experimentation in preclinical trials to predict evidences and perform the future translational researches.

**Abstract:**

Stem-cell therapy provides a promising strategy for patients with ischemic heart disease. In recent years, numerous studies related to this therapeutic approach were performed; however, the results were often heterogeneous and contradictory. For this reason, we conducted a systematic review and meta-analysis of trials, reporting the use of stem-cell treatment against acute or chronic ischemic cardiomyopathies in large animal models with regard to Left Ventricular Ejection Fraction (LVEF). The defined research strategy was applied to the PubMed database to identify relevant studies published from January 2011 to July 2021. A random-effect meta-analysis was performed on LVEF mean data at follow-up between control and stem-cell-treated animals. In order to improve the definition of the effect measure and to analyze the factors that could influence the outcomes, a subgroup comparison was conducted. Sixty-six studies (*n* = 1183 animals) satisfied our inclusion criteria. Ischemia/reperfusion infarction was performed in 37 studies, and chronic occlusion in 29 studies; moreover, 58 studies were on a pig animal model. The meta-analysis showed that cell therapy increased LVEF by 7.41% (95% Confidence Interval 6.23–8.59%; *p* < 0.001) at follow-up, with significative heterogeneity and high inconsistency (I^2^ = 82%, *p* < 0.001). By subgroup comparison, the follow-up after 31–60 days (*p* = 0.025), the late cell injection (>7 days, *p* = 0.005) and the route of cellular delivery by surgical treatment (*p* < 0.001) were significant predictors of LVEF improvement. This meta-analysis showed that stem-cell therapy may improve heart function in large animal models and that the swine specie is confirmed as a relevant animal model in the cardiovascular field. Due to the significative heterogeneity and high inconsistency, future translational studies should be designed to take into account the evidenced predictors to allow for the reduction of the number of animals used.

## 1. Introduction

Myocardial infarction is a leading cause of mortality and morbidity worldwide [1]. This pathology leads to death by necrosis of myocardial cells, due to prolonged ischemia, usually following coronary atherosclerosis [2]. In particular, coronary occlusion causes a loss of myocardial perfusion with consequent morphological, biochemical and functional alterations of the affected area, thus establishing ischemia, which, based on its extent and duration, can cause cell necrosis. Cell death leads to dramatic consequences because it triggers an acute inflammatory reaction. Subsequently, the damaged area is replaced by intensely vascularized granulation tissue, which then evolves into a process of fibrosis and, consequently, scar formation. Hyperplastic scar tissue is not functional, but the surviving patient’s heart must still find a way to function while maintaining adequate cardiac output. To do this, it undergoes a series of structural and dynamic changes which are referred to as “ventricular remodeling”. In fact, both the necrotic area and the non-infarcted segment of the ventricle progressively change in size, thickness and shape. All of this can then lead to heart failure [3]. Effective treatment strategies for myocardial infarction are designed to limit adverse ventricular remodeling to attenuate myocardial scar expansion and promote improvement of cardiac function and myocardial regeneration [4,5]. Among many therapies proposed, stem cells represent a promising option to repair the injured heart. Several cell types, including embryonic stem cells, skeletal myoblasts, mesenchymal stem cells (MSCs), cardiac stem cells (CSCs) and induced pluripotent stem cells (iPSCs), have been employed to re-functionalize the injured heart [5]. It has been shown that CSCs can differentiate into endothelial cells (ECs), vascular smooth-muscle cells (VSMCs) and cardiomyocytes (CMs) [6,7]. MSCs can differentiate into cardiomyocytes and induce angiogenesis [8,9]. Nevertheless, in vivo studies show that the percentage of inoculated stem cells that are stably implanted in the infarcted region and the related rate of cardiomyogenesis and angiogenesis are very slow to support myocardial regeneration [10]. However, studies from the past 20 years have clearly shown that it has been demonstrated that transplanted stem cells are able to release soluble factors that act in a paracrine way, contributing to the repair and regeneration of the infarcted myocardium [11]. These factors include a variety of growth factors, cytokines and extracellular matrix proteins [12]. Moreover, paracrine effects also include the recruitment activation and proliferation of resident endothelial progenitor cells (EPCs), cardiac progenitor cells (CPCs) and/or resident CSCs [12,13]. Furthermore, paracrine factors influence the contractile abilities of CM [6], promote cytoprotection (inhibition of apoptosis and necrosis) and formation of new blood vessels [7,12], prevent degradation of extracellular matrix (ECM), inhibit fibrosis and release of granulation factors [7]. Currently the most promising results have been obtained through the paracrine action rather than the direct action of cell differentiation [10,14,15,16].

In the last 20 years, in addition to the numerous experiments performed with in vitro models, numerous studies have been performed with large animal models with ischemic cardiomyopathies. These preclinical studies evaluated the risk of this new cell therapy, considering safety, feasibility and efficacy. In addition, they tried to answer the unsolved problems in clinical cell therapy (cell-type selection, number of cells, method of administration, time of administration and follow-up after cell transplantation); however, the results obtained were often heterogeneous and contradictory [17]. Studies based on large animal models often suffer from extremely limited sample sizes, due to ethical reasons, costs and management difficulties. Systematic reviews and meta-analysis substantially increase the statistical power, and the different experimental settings in the studies make it possible to obtain an estimate with a much higher external validity of the model. The present systematic review is an update of the previous work published by van der Spoel and colleagues [17] and aims to summarize trials reported in the literature about the use of stem-cell treatment against acute or chronic ischemic cardiomyopathies in large animal models and perform a meta-analysis on collected data from 2011 to 2021, with regard to Left Ventricular Ejection Fraction (LVEF) as a functional parameter.

## 2. Materials and Methods

### 2.1. Search Strategy and Selection Criteria

The international principles of preferred reporting items for systematic reviews and meta-analyses (PRISMA) 2020 guidelines were followed throughout the study [18]. Research was conducted in PubMed [19] to identify all the relevant publication from the period January 2011 to July 2021 by using the following search terms: “(pig OR porcine OR swine OR canine OR dog OR sheep OR ovine) AND (stem cells OR progenitor cells OR bone marrow) AND (myocardial infarction OR heart failure OR coronary artery disease OR cardiac repair OR myocardial regeneration)”. Only articles published in English were included. The collected studies were carefully examined, and duplicates were removed.

### 2.2. Eligibility Criteria

The primary literature used to conduct the systematic review was compliant with the following inclusion and exclusion criteria. Studies that used large animal models with acute myocardial infarction (MI) or chronic ischemic cardiomyopathies, randomized controlled trials (RCTs) or no RCT studies were included to investigate the effect of stem-cell therapy on cardiac function as determined by left ventricular ejection fraction (LVEF). In addition, a placebo or sham-operated control group was included in the study. Studies using reporter genes (for stem-cell-imaging purposes only) were also included. In vitro studies, studies using genetically engineered or transfected stem cells with altered cellular behavior and studies using only conditioned media were excluded. Reviews, editorials, comments, letters and reports were excluded.

### 2.3. Data Extraction

Two reviewers (D.L.M. and C.W.) independently selected the studies by reading titles, abstracts and full manuscripts and applying the criteria mentioned above, and the resulting list of studies was approved by a third reviewer (M.F.). Then the following information was extracted from the full text of the selected studies: basal characteristics of the studies and left ventricular ejection fraction (LVEF) outcomes. If necessary, LVEF data were recalculated as follows: (EDV − ESV)/EDV × 100% (EDV, end-diastolic volume, ESV, end-systolic volume). Accordingly, the standards deviations (SD) were determined or recalculated from the standard errors of mean (SEM).

### 2.4. Statistical Analysis

Primary analysis consisted of calculating the LVEF mean difference (reported in %) at follow-up between the control and stem-cell-treated groups when exposed to acute myocardial infarction or chronic ischemic cardiomyopathies. Continuous variables were reported as weighted mean differences with the 95% confidence intervals (CIs) between the treated and control groups. In the presence of multiple experimental groups alongside the control group within a study, the control group was used as control for each experimental group. A random-effect model (DerSimonian–Laird) was applied for the meta-analysis. Heterogeneity was assessed by using the I^2^ statistics. Values for 25%, 50% and 75% for I^2^ represented low, moderate and high heterogeneity, respectively [20]. In addition, the following subgroup analyses were performed: type of study (RCT or cohort); MI model (ischemia/reperfusion (I/R) or chronic occlusion); location of infarct-related artery (left anterior descending artery (LAD) or left circumflex artery (LCX)); autologous cell therapy (yes or no); cell type (adipose-derived stem cells (ASCs), bone-marrow mononuclear cells (BMMNC), bone-marrow-derived mononuclear cells (BMDMNCs), bone-marrow stem cells (BMSCs), cardiosphere-derived cells (CDCs), cardiac-derived progenitor cells (CPCs), cardiac stem cells (CSCs), multipotent adult progenitor cells (MAPCs), mesenchymal precursor cells (MPCs), induced pluripotent stem cells (iPSCs), mesenchymal stem cells (MSCs) or other types of stem cells (SC)); number of cells injected (<10^7^, 10^7^–10^8^, ≥10^8^); timing of cell therapy after heart attack (<1 day, 1–7 days, >7 days); follow-up after cell therapy (≤30 days, 31–60 days, >60 days); type of animal (pig, dog or sheep); and route of delivery (intramyocardial (IM), intracoronary (IC), trans-endocardial (TE), surgical or other routes)). Welch’s *t*-test or ANOVA test was applied to compare subgroups. A funnel plot for LVEF was drawn to explore publication bias. All analyses were performed by using JASP software (JASP Team, 2022; version 0.14.1; Amsterdam, The Netherlands).

## 3. Results

### 3.1. Study Selection

**Figure 1:**
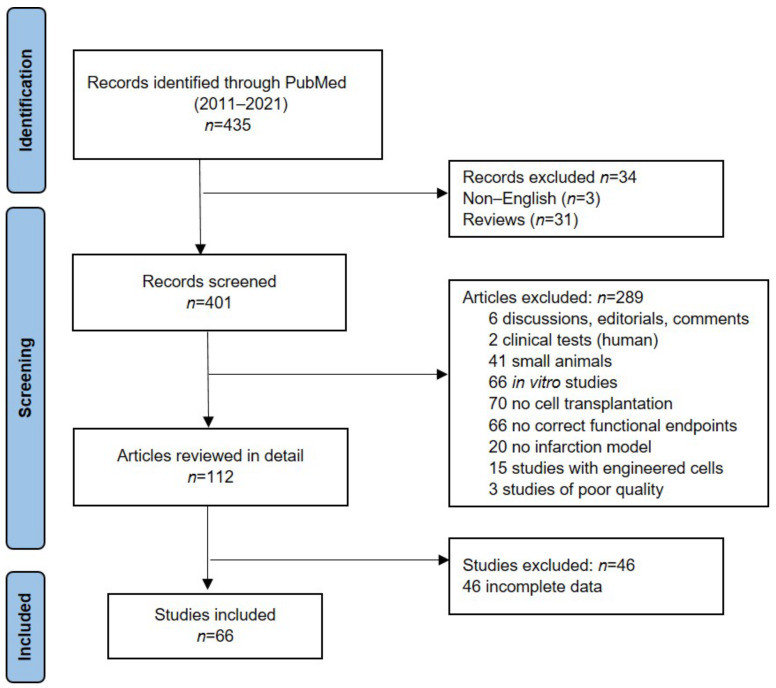
PRISMA workflow of the study selection process, records screened and studies included.

The database search yielded 435 publications. After removing articles not in English and reviews, 401 publications were identified and assessed for eligibility. Based on the defined criteria, 289 studies were excluded and 112 studies were reviewed in detail. Only 66 studies met our inclusion criteria. The study search and selection processes are described in detail in Figure 1.

### 3.2. Included Studies Characteristics

In total, 1183 animals met the inclusion criteria, and the data derived from them were analyzed. Table 1 provides the characteristics of the included studies. Most studies used the porcine model (58 studies). In 37 studies, ischemia/reperfusion was used as an MI model. MI was mainly induced in the LAD (60 studies), but the site of ligation/constriction of the vessel (proximal, mid or distal) varied. Ten different types of cells were studied (25 studies used MSCs), but the number of stem cells administered varied (from 10^6^ to 10^9^); 26 studies used autologous cells. The main routes of delivery were IC infusion, IM, TE injection and surgical. Cell therapy was performed at different times after MI: <1 day (21 studies), 1–7 days (12 studies) and >7 days (33 studies). Follow-ups after cell therapy varied from 1 day to 180 days. The median and interquartile range of follow-up time was 51 days (28–60 days).

### 3.3. Meta-Analysis

**Figure 2:**
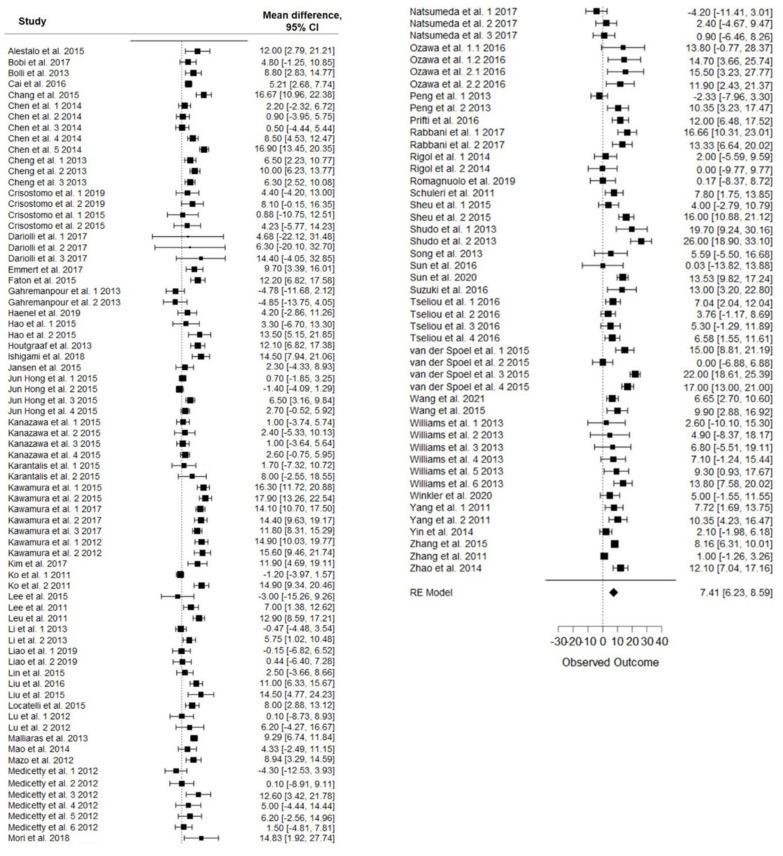
Forest plot showing the effect of stem-cell therapy on LVEF improvement compared with controls. Note: 95% CI, 95% confidence interval.

Meta-analysis showed a LVEF difference of 7.41% at follow-up after stem-cell therapy vs. control (95% CI, 6.23–8.59%; *p* < 0.001), with significative heterogeneity (*p* < 0.001) and high inconsistency (I^2^: 82%) (Figure 2). At follow-up, the mean LVEF after stem-cell treatment and control was 48% and 40.7%, respectively.

### 3.4. Subgroup Analysis

**Figure 3:**
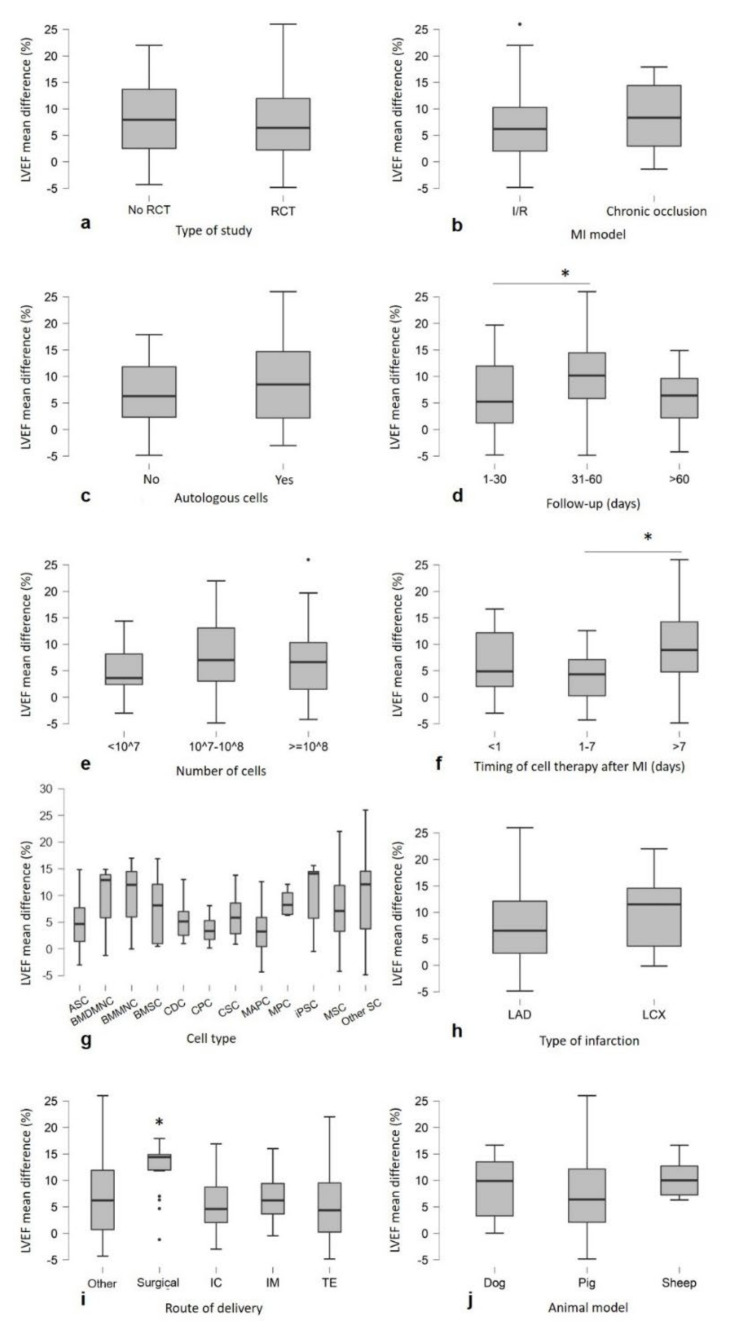
Subgroup analysis showing the LVEF trends toward more improvements after cell therapy compared with control: (**d**) follow-up at 31–60 days (*p* = 0.025), (**f**) the late cell injection (>7 days, *p* = 0.005), (**i**) surgical administration (*p* < 0.001), (**b**) chronic occlusion model (*p* = 0.063) and (**c**) autologous cells (*p* = 0.079). No significant differences were observed in (**j**) animal model (*p* = 0.355), (**h**) type of infarction (*p* = 0.257), (**a**) type of study (*p* = 0.345), (**e**) number of cells (*p* = 0.39) and (**g**) cell type (n ≥ 3 studies) (*p* = 0.361). Graphs are represented as Boxplots; the two segments that delimit the rectangle represent the 25th and 75th percentiles; the central segment is the median; the bars represent the minimum and maximum values, respectively; and the external points are the outliers. Note: (* *p* <0.05) represents statistical significance resulting from one-way ANOVA, followed by post hoc Tukey comparison test.

**Figure 4:**
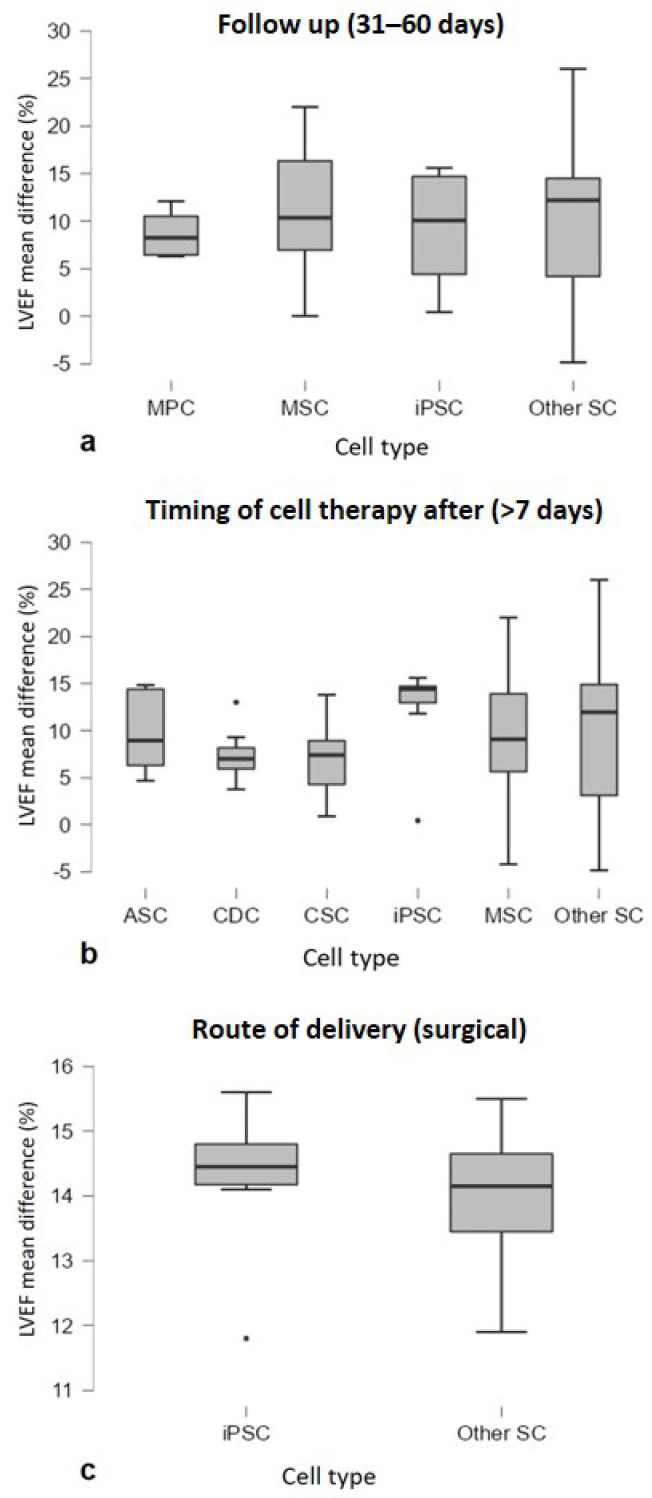
Subgroup analysis showing LVEF trends of cell type (*n* > 3 studies) by considering studies of the three significant predictors, in particular (**a**) follow-up (30–60 days) (*p* = 0.904), (**b**) timing of cell therapy after MI (>7 days) (*p* = 0.690) and (**c**) route of delivery (surgical) (*p* = 0.729). Graphs are represented as Boxplots; the two segments that delimit the rectangle represent the 25th and 75th percentiles; the central segment is the median; the bars represent the minimum and maximum values, respectively; and the external points the outliers.

LVEF mean difference values were compared for subgroup analysis, and a Welch’s *t*-test or ANOVA test was applied. The analysis showed that follow-up after cell therapy (*p* = 0.025), time between infarction and cell injection (*p* = 0.005) and the route of delivery (*p* < 0.001) are independent significant predictors of LVEF improvement. Figure 3d,f,i shows a trend toward greater improvements after cell therapy in the following aspects: follow-up at 31–60 days, since, after that period, the effect of cell therapy appeared to decline over time; and the late cell injection after MI (>7 days) and the surgical treatment. In addition, less benefit was observed in the ischemia/reperfusion MI model compared to the chronic MI models (*p* = 0.063), and there was an improvement with autologous cell treatment (*p* = 0.079) (Figure 3b,c). No significant differences in LVEF were observed in the following cases: animal model (*p* = 0.355), type of infarction (*p* = 0.257), type of study (*p* = 0.345), cell number (*p* = 0.39) and cell type (*p* = 0.361) (Figure 3a,e,g,h,j). An additional subgroup analysis was performed to analyze the three predictors at the significant levels (Follow up 31–60 days, timing of cell therapy after MI > 7 days and surgical as route of delivery) to understand whether the effect on LVEF improvement was related to a specific cell type. No significative differences were detected (Figure 4).

**Figure 5:**
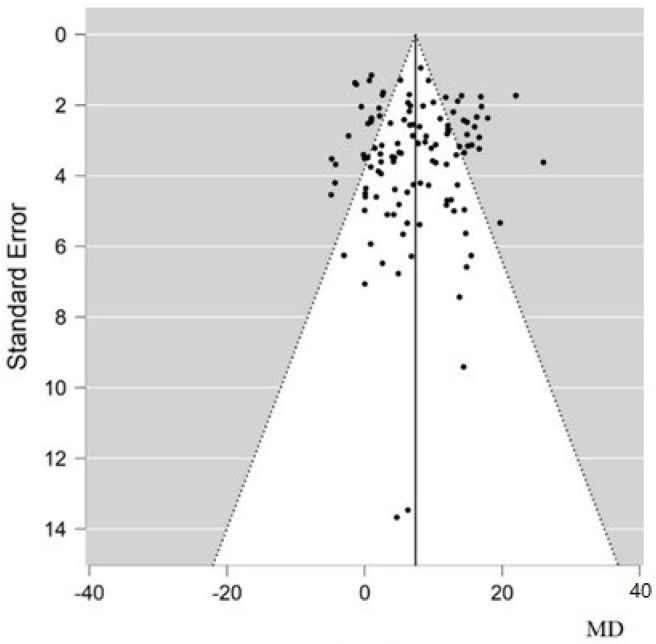
Funnel plot for LVEF improvement showing the absence of publication biases. The vertical solid line represents the estimated overall mean difference; black dots are the standard error of each study. MD, mean difference.

The funnel plot for LVEF mean difference shows that there is no publication bias (Figure 5), as values are evenly distributed around the effect estimate, as evidenced by the regression test for funnel plot asymmetry (Egger’s test) (*p* = 0.657).

## 4. Discussion

In the present systematic review and meta-analysis, we assessed the effect of stem-cell therapy against ischemic cardiomyopathies in large animal models; this is an update of a previous work published by van der Spoel et al. [17] that reviewed the same topic in studies performed from 1980 to 2010. The analysis includes data from 66 published pre-clinical studies (2011–2021) that used large animal models treated with stem cells in order to study the effects of cell therapy of ischemic cardiomyopathies by reporting outcomes derived from left ventricular ejection fraction (LVEF) as functional parameter. The meta-analysis showed a significant improvement of LVEF by 7.41% (95% CI 6.23–8.59%) after stem-cell therapy against control group confirming the positive effect reported in the previous meta-analysis [17], in which LVEF effect size was 7.51% (95% CI 6.15–8.87%). Given the number of the studies included, a random-effect model was applied by resulting a significative heterogeneity with high inconsistency (I^2^: 82%). For this reason, a comparison between subgroups was investigated in order to analyze clinically relevant parameters. The sub-analysis revealed that time of follow-up, time between infarction and cell injection, and the route of cellular delivery are independent significant predictors of LVEF improvement. In detail, in large animals, the effect of cell therapy achieved better results at 31–60 days, after which it fades over time; this phenomenon is in accordance with the previous analysis [17]. This finding could suggest the use of new applications and therapeutic strategies to increase cell survival over time, such as the use of slow-release molecules by cell pre-conditioning [86,87], the application of biomaterials [88,89] or the genetic stem-cell modifications [90]. Late cell injection assumed better benefit if applied 7 days after MI; our findings are comparable with the previous meta-analyses both in large animals [17] and human [91]. Optimal stem-cell therapy depends not only on engraftment and survival of the transplanted cells but also on successful delivery. By comparing different types of cellular delivery, our results demonstrated that surgical treatment is the route that significantly improves the heart functionality. In general, stem cells can be delivered by intravenous or intracoronary routes after coronary revascularization in the setting of acute MI to avoid the risk of invasive procedure; however, both IV and IC routes seems to be not applicable for patients with chronic myocardial ischemia not amenable to coronary revascularization, so direct intramyocardial injection via either surgical epicardial or transcatheter endocardial approaches may be necessary, as they allow for the direct visualization of the site of injection [92]. In addition, our findings showed that less benefit in LVEF improvement was observed in ischemia/reperfusion MI model compared to the chronic occlusion models, but without reaching significance. This is compliant with the findings obtained by van der Spoel et al. [17] both in large and small animal models [93]. Autologous cell therapy resulted in better results on LVEF improvement, but not significative; a similar effect was shown in a meta-analysis performed in large animal models about autologous and allogeneic cell therapy for ischemic heart disease by using BM-MNCs, MSCs and cardiac stem-cell types [38]. Furthermore, the use of autologous BM- or MSC-derived cells is confounded by the functional impairment of those stem cells associated with aging and because of the restricted immediate availability; the use of allogenic cell products with limited immunogenicity, such as MSC derived from different tissues, or standardized non-cellular products, such as conditional medium, may overcome these problems in terms of efficacy and safety [38,92,94].

No significative differences in LVEF were observed in animal species, infarct type, type of study, number of cells and cell type. Regarding the cell type, the result obtained contrasts with that of van der Spoel et al. [17]. We could not exclude that this result is due to the high difference in the studies’ number between the group analyzed. The same consideration regarding the lack of significative results deserves to be made for the number of cells (<10^7^ *n* = 6, 10^7^–10^8^ *n* = 41, and ≥ 10^8^ *n* = 19) and the animal species (porcine *n* = 58 others *n* = 8).

Although no difference was observed between species, we would sustain the widely accepted porcine animal model as the one to be recommended to evaluate the effect of the cell therapy, confirming the swine as a relevant animal model in the cardiovascular field and in translational research in a broader sense [95]. This is because most of the published studies (89% of those included in our SR) are based on this model, and the data are, therefore, available for comparison as a fundamental tool in future experimental designs, in particular, in relation to the Reduction aspects. Furthermore, we wanted to investigate what could contribute to the statistical significance of the improving predictors of LVEF. In particular, we analyzed whether the effect of LVEF improvement was attributed to a specific cell type. No significative difference was observed in cell type in large animal studies with a significative improvement in follow-up (31–60 days), timing of cell therapy after MI (>7 days) and surgical treatment (Figure 4).

### Limitations

The limitations of meta-analysis are well-known [96]. Meta-analyses and systematic reviews are statistical and scientific techniques that can highlight areas where evidence is lacking, but they cannot overcome these deficiencies [97]. Publication bias and search bias are potential problems in all meta-analyses [97]; this arises from the fact that unpublished studies may contradict the results due to the tendency not to publish negative studies, thus leading to the over-representation of “positive” ones [98]. In this meta-analysis, the funnel plot (Figure 4) for the LVEF mean difference showed that there is no publication bias. Thus, based on the results we obtained, we can affirm that, in the future, stem-cell-transplant studies in large animal models with ischemic cardiomyopathies should therefore focus on late (>7 days) surgical treatments and 31–60-day follow-up. The analysis of the subgroups shows that the greater heterogeneity of the included studies could be mainly due to the different amounts of data in the different comparison groups, such as in the case of cell number, cell type and animal species.

## 5. Conclusions

In conclusion, in the present systematic review and meta-analysis, we evaluated the effect of stem-cell transplantation in large animal models with ischemic cardiomyopathies, showing that stem-cell therapy could improve LVEF. The SR is therefore confirmed as a reliable method for obtaining a complete contextual framework from which to start for further experimentation, and future translational studies should be designed by considering the evidenced predictors to allow for the reduction of the number of animals used in preclinical trials. Large animal models, especially the swine, are a translational step necessary to predict outcomes of clinical trials in the cardiovascular field.

## Figures and Tables

**Table 1:** Characteristics of the included studies.

Author	*n*	Type of Animal	Type of Study	Type of Infarction	MI Model	Cell Type	Number of Cells	Autologous Cells (Yes or No)	Route of Delivery	Timing of Cell Therapy after MI ^a^	Follow-Up (Days)
Alestalo et al. 2015 [21]	24	Pig	RCT	LCX	I/R	BMMNC	6.2 × 10^7^–1.43 × 10^8^	Yes	Surgical	1.5 h	21
Bobi et al. 2017 [22]	14	Pig	RCT	LAD	I/R	ATMSC	1 × 10^7^	No	IC	1 h	60
Bolli et al. 2013 [23]	21	Pig	No RCT	LAD	I/R	CSC	5 × 10^5^	Yes	IC	90 d	30
Cai et al. 2016 [24]	20	Pig	RCT	LAD	No I/R	BMMSC	3 × 10^7^	Yes	IM	0.5 h	28
Chang et al. 2015 [25]	12	Dog	RCT	LAD	No I/R	BMSC	2 × 10^7^	Yes	IC	4 h	28
Chen et al. 2014 [26]	50	Pig	RCT	LAD	No I/R	BMSC	1 × 10^7^	Yes	IC	3 h or 1 d or 3 d or 7 d or 14 d	28
Cheng et al. 2013 [27]	39	Sheep	RCT	LAD	I/R	MPC	2.5 × 10^7^, 7.5 × 10^7^, 2.25 × 10^8^	No	TE	28 d	56
Crisostomo et al. 2019 [28]	25	Pig	RCT	LAD	I/R	CPC	2.5 × 10^7^, 5.0 × 10^7^	No	IC	7 d	70
Crisostomo et al. 2015 [29]	17	Pig	No RCT	LAD	I/R	CSC	2.5 × 10^7^	No	IC	2 h or 7 d	70
Dariolli et al. 2017 [30]	25	Pig	RCT	LCX	No I/R	pASC	1 × 10^6^, 2 × 10^6^, 4 × 10^6^	No	Surgical	30 d	30
Emmert et al. 2017 [31]	18	Pig	RCT	LAD	I/R	cardiopoietic stem cells	5 × 10^7^	No	IM	30 d	30
Fanton et al. 2015 [32]	18	Pig	RCT	LAD	I/R	CASC	8.3 × 10^7^ ± 1.26 × 10^8^	Yes	IM	2 h	60
Gahremanpour et al. 2013 [33]	30	Pig	RCT	LAD	I/R	USSC	3.02 × 10^8^ ± 2.3 × 10^7^	No	TE	10 d	28–56
Haenel et al. 2019 [34]	17	Pig	RCT	LAD	I/R	ADRC	1.8 × 10^7^	Yes	RCV	28 d	42
Hao et al. 2015 [35]	12	Dog	RCT	LAD	I/R	MSC	1 × 10^7^	No	IC	2–3 h	70
Houtgraaf et al. 2013 [36]	34	Sheep	RCT	LAD	I/R	MPC	1.25 × 10^7^–3.75 × 10^7^	No	IC	1.5 h	56
Ishigami et al. 2018 [37]	10	Pig	RCT	LAD	No I/R	hiPSC	1 × 10^7^	No	Surgical	14 d	28
Jansen of Lorkeers et al. 2015 [38]	16	Pig	RCT	LAD	I/R	hCMPC	1 × 10^7^	No	IC	28 d	28
Jun Hong et al. 2015 [39]	21	Pig	RCT	LAD	No I/R	ASC	1.5 × 10^8^, 5 × 10^7^×3	No	IV	1 h	2–28
Kanazawa et al. 2015 [40]	14	Pig	RCT	LAD	I/R	CDC	5 × 10^6^, 7.5 × 10^6^, 1 × 10^7^, 8.7 × 10^6^	No	IC	0.5 h	2
Karantalis et al. 2015 [41]	20	Pig	RCT	LAD	I/R	MSC/MSC+CSC	2 × 10^8^/2 × 10^8^+1 × 10^6^	Yes	TE	90 d	90
Kawamura et al. 2015 [42]	12	Pig	RCT	LAD	No I/R	BMMSC	1 × 10^8^	No	Surgical	28 d	28–56
Kawamura et al. 2017 [43]	11	Pig	RCT	LAD	No I/R	hiPS-CM	3.5 × 10^7^	No	Surgical	28 d	30–60–90
Kawamura et al. 2012 [44]	12	Pig	RCT	LAD	No I/R	hiPS-CM	3.2 × 10^7^	No	Surgical	28 d	28–56
Kim et al. 2017 [45]	18	Pig	No RCT	LAD	No I/R	ATMSC	1 × 10^7^	No	percutaneous	7 d	21
Ko et al. 2011 [46]	12	Pig	RCT	LAD	No I/R	BMDMNC	3 × 10^7^	Yes	Surgical	0.25 h	3–90
Lee et al. 2015 [47]	28	Pig	No RCT	LAD	I/R	ADSC	2 × 10^6^	Yes	IC	0.5 h	28
Lee et al. 2011 [48]	21	Pig	RCT	LAD	I/R	CDC	1 × 10^7^	Yes	Surgical	28 d	56
Leu et al. 2011 [49]	12	Pig	No RCT	LAD	No I/R	BMDMNC	3 × 10^7^	Yes	Surgical	Immediately	90
Li et al. 2013 [50]	24	Pig	RCT	LAD	No I/R	iPS	2 × 10^7^	No	IM	7 d	7–42
Liao et al. 2019 [51]	24	Pig	RCT	LCX	No I/R	CM/MSC	2 × 10^8^/2 × 10^8^	No	IM	56 d	56
Lin et al. 2015 [52]	10	Pig	No RCT	LAD	No I/R	MNC	1 × 10^8^	Yes	IM	Immediately	90
Liu et al. 2016 [53]	12	Pig	RCT	LCX	No I/R	UC-MSC	3 × 10^7^ + 3 × 10^7^	No	IC+IV	28 d + 35–42 d	28
Liu et al. 2015 [54]	12	Pig	No RCT	LAD	No I/R	PDMC	1 × 10^7^	No	Surgical	Immediately	56
Locatelli et al. 2015 [55]	16	Sheep	RCT	LAD	No I/R	MSC	2 × 10^7^	No	Intramyocardial transepicardial	7 d	30
Lu et al. 2012 [56]	24	Pig	RCT	LAD	I/R	MSC	3 × 10^7^	Yes	IC	7 d	3–42
Malliaras et al. 2013 [57]	10	Pig	RCT	LAD	I/R	CDC	1.25 × 10^7^	No	IC	14–21 d	60
Mao et al. 2014 [58]	16	Pig	RCT	LAD	I/R	MSC	1.5 × 10^7^	No	IM	7 d	28
Mazo et al. 2012 [59]	16	Pig	RCT	LAD	I/R	ADSC	2.1 × 10^8^ ± 4.2 × 10^7^	Yes	Percutaneous myocardial	9 d	90
Medicetty et al. 2012 [60]	19	Pig	No RCT	LAD	I/R	MAPC	2 × 10^7^, 2 × 10^8^	No	Percutaneous adventitial	2 d	2–30–90
Mori et al. 2018 [61]	12	Pig	RCT	LAD	No I/R	ADSC	1 × 10^8^	No	Cell spray	28 d	28
Natsumeda et al. 2017 [62]	25	Pig	RCT	LAD	I/R	MSC/CSC/MSC+CSC	2 × 10^8^/1 × 10^6^/2 × 10^8^ + 1 × 10^6^	No	TE	90 d	90
Ozawa et al. 1 2016 [63]	10	Juvenile pig	No RCT	LAD	I/R	SSC	4.5 × 10^7^−6 × 10^7^	Yes	Surgical	28 d	28–56
Ozawa et al. 2 2016 [63]	10	Adult Pig	No RCT	LAD	I/R	SSC	1.5 × 10^8^	Yes	Surgical	28 d	28–56
Peng et al. 2013 [64]	10	Pig	RCT	LAD	I/R	MSC	1 × 10^8^−2.3 × 10^8^	Yes	IC	7–14 d	7–56
Prifti et al. 2016 [65]	25	Pig	No RCT	LAD	I/R	Mouse skeletal C2C12 myoblasts	NA	No	Venous coronary sinus retrograde infusion	30 d	30
Rabbani et al. 2017 [66]	18	Sheep	No RCT	LAD	No I/R	MSC/EC	2.7 × 10^7^	Yes	Surgical	Immediately	60
Rigol et al. 2014 [67]	24	Pig	No RCT	LAD	I/R	ATMSC	1 × 10^7^	No	IC	0.25 h or 7 d	21
Romagnuolo et al. 2019 [68]	10	Pig	No RCT	LAD	I/R	hESC-CM	1 × 10^9^	No	Transepicardial	21 d	28
Schuleri et al. 2011 [69]	22	Pig	RCT	LAD	I/R	MSC	2 × 10^8^	No	IM	84 d	84
Sheu et al. 2015 [70]	12	Pig	No RCT	LAD	No I/R	BMMSC	3 × 10^7^	Yes	IM	1 h	4–60
Shudo et al. 2013 [71]	12	Pig	RCT	LAD	I/R	SMB	4.5 × 10^8^	Yes	Cell sheets transepicardial	28 d	28–56
Song et al. 2013 [72]	14	Pig	RCT	LAD	I/R	BMMSC	3 × 10^7^	Yes	IM	2 h	28
Sun et al. 2016 [73]	14	Dog	RCT	LAD	No I/R	MSC	1 × 10^7^	Yes	RCV	7 d	40
Sun et al. 2020 [74]	16	Pig	RCT	LCX	No I/R	hiPSC-MSC	2 × 10^8^	No	IM	56 d	56
Suzuki et al. 2016 [75]	11	Pig	RCT	LAD	No I/R	CDC	2 × 10^7^	No	IC	60 d	28
Tseliou et al. 1 2016 [76]	15	Pig	RCT	LAD	No I/R	CDC	1.25 × 10^7^	No	Single-vessel intracoronary (stop-flow or continuous-flow)	21 d	28
Tseliou et al. 2 2016 [76]	15	Pig	RCT	LAD	No I/R	CDC	1.25 × 10^7^	No	Multi-vessel intracoronary (stop-flow or continuous-flow)	21 d	28
van der Spoel et al. 2015 [77]	17	Pig	No RCT	LCX	I/R	MSC/BMMNC+MSC	1 × 10^7^/1 × 10^7^ + 1 × 10^7^	Yes	TE	28 d/28 d + 56 d	28–56
Wang et al. 2021 [3]	30	Pig	No RCT	LAD	I/R	MSC	1 × 10^8^	No	IM	60 d	90
Wang et al. 2015 [78]	8	Dog	RCT	LAD	No I/R	MSC	1 × 10^8^	No	RCV	7 d	28
Williams et al. 2013 [79]	20	Pig	No RCT	LAD	I/R	MSC/CSC/MSC+CSC	2 × 10^8^/1 × 10^6^/2 × 10^8^ + 1 × 10^6^	No	IM	14 d	14–28
Winkler et al. 2020 [80]	13	Pig	RCT	LAD	I/R	CDC	1 × 10^7^	No	IC	0.25 h	30
Yang et al. 2011 [81]	25	Pig	RCT	LAD	I/R	MSC	9 × 10^7^−1.8 × 10^8^	No	IC	14 d	42
Yin et al. 2014 [82]	10	Pig	RCT	LAD	I/R	ASC	4 × 10^7^	Yes	IC	7 d	56
Zhang et al. 2015 [83]	12	Pig	RCT	LAD	No I/R	BMSC	2 × 10^7^	Yes	IM	NA	42
Zhang et al. 2011 [84]	12	Pig	RCT	LAD	No I/R	BMSC	2 × 10^7^	Yes	IM	NA	42
Zhao et al. 2014 [85]	20	Pig	RCT	LAD	No I/R	BMSC	1 × 10^7^	NA	IM	Immediately	180

ADRC, adipose-derived regenerative cells; ADSC, adipose tissue-derived stem cells; AMSC, amniotic-membrane-derived mesenchymal stromal cell; ASC, adipose-derived stem cells; ATMSC, adipose tissue–derived mesenchymal stem cells; BMDMNC, bone-marrow-derived mononuclear cell; BMSC, bone-marrow stem cells; BMMSC, bone-marrow mesenchymal stem cells; BM-MNC, bone-marrow mononuclear cells; CASC, cardiac atrial appendage stem cells; CB-MNC, human cord blood mononuclear cells; CBSC, cortical-bone stem cells; CDC, cardiosphere-derived cells; CPC, cardiac-derived progenitor cells; CSC, cardiac stem cells; EC, endothelial cells; EPC, endothelial progenitor cells; ESC, embryonic stem cells; hCMPC, human cardiomyocyte progenitor cells; hESC-CM, human embryonic stem-cell-derived cardiomyocytes; hiPSC-MSC, human-induced pluripotent stem cell-derived mesenchymal stem cells; hiPSC, human-induced pluripotent stem cell; hiPS-CM, human-induced pluripotent stem-cell-derived cardiomyocytes; HPC, hematopoietic progenitor cells; iPS, induced pluripotent stem cells; IC, intracoronary infusion; IM, intramyocardial injection; I/R, ischemia/reperfusion; IV, peripheral intravenous; LAD, left anterior descending artery; LCX, left circumflex artery; MAPC, multipotent adult progenitor cells; MI, myocardial infarction; MNC, mononuclear cells; MPC, mesenchymal precursor cells; MSC, mesenchymal stem cells; *n*, number of animals (treated group and control group); NA, not applicable; pASC, porcine-adipose-tissue-derived mesenchymal stem cells; PDMC, placenta-derived multipotent cells; RCT, randomized controlled trail; RCV, retrograde coronary transvenous injection; SMB, skeletal myoblast; SSC, skeletal stem cells; TE, trans-endocardial injection; UC-MSC, umbilical-cord-derived mesenchymal stromal cells; USSC, unrestricted somatic stem cells. ^a^ Timing in hours (h) or days (d).

## Data Availability

Not applicable.
